# A Country Doctor’s Experience with Antipyretics

**Published:** 1891-08

**Authors:** R. S. Gregg

**Affiliations:** Manor, Texas


For Daniel's Texas Medical Journal.

### A COUNTRY DOCTOR'S EXPERIENCE WITH ANTI-PYRETICS.

BY R. S. GREGG, M. D., MANOR, TEX.

[Read at Austin District Medical Society.]

HAVING been requested by our worthy secretary to write an article on antipyretics, I will endeavor to give this society my experience with such of these agents as I have had sufficient confidence in to dispense to my patients.

Much has recently been written on antipyretics. Nearly all the articles which I have had the pleasure of reading, have had very little to say about the "modus operandi" of these remedies, but only give their effects. Theoretically, I believe, they are supposed to act upon the nerve centers, especially the "heat center." Of course, this theory, like many others cannot be very well demonstrated. The effects of these drugs we can plainly recognize, and that they are very potent no one who has used them will deny. My experience has been with only a few of the many that have been given to the profession during the last five or six years. Antipyrin, antifebrin and phenacetin are the antipyretics that I am now, and have been using in my practice for several years. In talking with my medical brethren, I find much difference of opinion with regard to the effects of these agents. As this paper is intended to give my personal experience with each of them, I will give no hearsay evidence.

I think I have used antipyrin and antifebrin in about an equal number of cases. I have used them in malarial diseases, pneumonia, diphtheria, phthisis, and in short, whenever I thought the high temperature was likely to do more harm than the remedies; for I must say, gentlemen, that I am convinced that we are only left to choose between the lesser of two evils, these coal-tar preparations, or an excessive, prolonged, high temperature.

If we have a sudden, high rise of temperature, in a robust, strong patient, from a malarial fever, sun-stroke, or any acute inflammatory trouble, we can confidently expect to relieve our patient with an appropriate dose of these antipyretics. But if our patient has been struggling with any of the exhausting maladies, such as typhoid fever, diphtheria, in fact, all such diseases as tend to death by exhaustion, we should use such remedies cautiously, if at all. We have had in our village during the last two months some eight or ten cases of typhoid fever, and it has fallen to my lot to treat several of them. In nearly all these cases I thought the high temperature called for an antipyretic. In several instances when I gave antifebrin, I had cause to regret it. In three of my cases the temperature was reduced to 97°, and my patient was covered with a cold perspiration, the pulse becoming exceedingly weak and rapid. Cyanosis was produced to an alarming extent in one case. I have never observed so much depression from antipyrin. Both act very freely on the skin, but antifebrin certainly has a much more depressing effect upon the circulation. It is becoming quite fashionable for persons, nervous, delicate ladies especially, who suffer from headaches, produced by whatever causes, to keep these drugs on hand and dose themselves with them. It is our duty as physicians to inform such persons that they are tampering with dangerous drugs.

Phenacetin I have used occasionally as an antipyretic, but more often as an analgesic. It certainly relieves pain more promptly, but as antipyretic, I cannot see that it has any advantage over antipyrin. It acts equally as much on the skin, and my patients complain of its depressing effects in about the same proportion. I notice that some physicians are prescribing quinine and one of these antipyretics in combination. In our continued fevers I do not like this mixture of the two drugs, to be repeated every two, three or four hours. It may bring about more depression than we expect, and the effect of the antipyretic may deceive us as to the course of the fever. After giving such a mixture, if a country pdysician rides eight or ten miles to see his patient and finds the temperature down to 100°, or even normal, and tells his patient that it will not be necessary to repeat his visit, he may feel rather chagrined to hear in a day or two that a brother physician has been called to the case that he cured.

I now come to the antipyretic par excellence, which is cold water. I believe we have no agent which will reduce a high temperature so promptly, and with so little deleterious effect to our patients. I know there are some who think that this rapid cooling of the surface will induce a congestion of the important internal organs, but such has not been my experience. I have never had cause to regret the use of the cold bath. I have often put my patient, with a temperature of from 106° to 108°, in cold water, and have always found the temperature rapidly reduced, and have yet to regret doing so. Only a few days ago I was called to a Mexican child aged two years. The child had a chill about 12 o'clock, and when I arrived, about 3 o'clock, was having convulsions, and temperature in axilla registered 107¾°. I immediately put him in a tub of tepid water, and commenced pouring cold water fresh from the well, over his head, allowing it to run down into the tub until I had used four or five water-bucketsful, and had the water in tub about as cold as the well water. He was in the water fifteen or twenty minutes, when I took him out and wrapped him in a woolen shawl. Convulsions had ceased, and entire surface felt cold. I took his temperature in rectum and found it 101½°. Such has been my experience in treating high temperatures in many of my little patients, and I only regret that the proper conveniences are so often wanting in a country practice, for treating adults in the same manner.

